# Human macrophage pro‐inflammatory polarization in response to free cholesterol and cholesterol remnants

**DOI:** 10.14814/phy2.70367

**Published:** 2025-05-22

**Authors:** Karel Paukner, Barbora Muffova, Hana Bartuskova, Jan Mareš, Libor Janousek, Jiri Fronek, Sona Kauerova, Ivana Kralova Lesna, Rudolf Poledne

**Affiliations:** ^1^ Laboratory for Atherosclerosis Research Centre for Experimental Medicine, Institute for Clinical and Experimental Medicine Prague Czech Republic; ^2^ Department of Physiology, Faculty of Science Charles University Prague Czech Republic; ^3^ Department of Data Science Institute for Clinical and Experimental Medicine Prague Czech Republic; ^4^ Transplant Surgery Department Institute for Clinical and Experimental Medicine Prague Czech Republic

**Keywords:** adipose tissue, atherosclerosis, cholesterol/cell and tissue, inflammation, lipoproteins

## Abstract

Atherosclerosis is a chronic inflammatory disease of the blood vessels caused by elevated levels of lipoproteins. The hyperlipoproteinemia triggers a series of cellular changes, particularly the activation of the macrophages, which play a crucial role in the development and progression of atherosclerosis. The presence of free cholesterol (FC) in lipoproteins may contribute to macrophage stimulation. However, the mechanisms linking the accumulation of FC in macrophages to their pro‐inflammatory activation remain poorly understood. Our research found a positive correlation between the number of pro‐inflammatory macrophages (CD14 + CD16 + CD36^high^) in visceral adipose tissue and the levels of LDL‐C and cholesterol remnant particles in 56 healthy people. In contrast, the proportion of anti‐inflammatory, alternatively activated macrophages (CD14 + CD16‐CD163+) correlated negatively with HDL‐C. Additionally, our in vitro study demonstrated that macrophages accumulating FC promoted a pro‐inflammatory response, activating the *TNF‐α* and chemokine *CCL3* genes. Furthermore, the accumulation of FC in macrophages alters the surface receptors on macrophages (CD206 and CD16) and increases cellular granularity. Notably, the CD36 surface receptor and the *ACAT* and *CD36* genes did not show a response. These results suggest a link between excessive FC accumulation and systemic inflammation to underlie the development of atherosclerosis.

## 1 INTRODUCTION

Atherosclerosis, a pathology responsible for the majority of deaths worldwide, involves an interplay between increased levels of cholesterol‐carrying lipoproteins and the immune system within the arterial wall. The substantial role of cholesterol molecules in atherosclerotic cardiovascular disease (ASCVD) has been described (Brewer Jr., 2011). Even though cholesterol is primarily present as cholesterol esters in living cells and circulating lipoproteins, the potential impact of free cholesterol on overall body turnover of cholesterol should not be underestimated (Jakulj et al., 2016). While it is widely recognized that high serum cholesterol levels are associated with the buildup of atherosclerotic lesions, our understanding of the underlying mechanisms continues to evolve. An area currently receiving considerable research attention is the role of remnant particles and free cholesterol, not only in the development of ASCVD but also in influencing the phenotype of macrophages, which are critical cells in the progression of atherosclerosis (Cordero et al., 2023; Wadström et al., 2023). Our previous results suggest that non‐HDL cholesterol concentrations positively correlate with the proportion of pro‐inflammatory macrophages within the visceral adipose tissue of healthy humans (Poledne et al., 2016).

Lipoproteins generally play an important role in transporting cholesterol to and from tissues. The plasma levels of LDL‐C and HDL‐C are commonly measured to predict the risk of atherosclerosis development. Though most useful and reliable, these traditional biomarkers do not always entirely reflect the risk of cardiovascular events. It has been recognized that even after lowering LDL‐C to the recommended goal, residual ASCVD risk remains (Jepsen et al., 2016) thus, the term “residual risk” strongly suggests that other players modulating systemic cholesterol metabolism may also contribute to cardiovascular disease risk (Ridker et al., 2010). The residual risk could partly be due to remnant cholesterol in triglyceride‐rich lipoproteins associated with the increased risk of ASCVD seen in genetic, observational, and clinical intervention studies (Castañer et al., 2020; Jepsen et al., 2016; Langsted et al., 2020; Varbo et al., 2013; Wadström et al., 2023). Remnant cholesterol, also called triglyceride‐rich lipoprotein cholesterol, includes the cholesterol in very low‐density lipoproteins (VLDL), intermediate‐density lipoproteins (IDL), and chylomicron remnants in the non‐fasted state (Stürzebecher et al., 2023).

Alongside the role of remnant particles, the impact of free cholesterol is also under investigation (Gerl et al., 2018). Recent studies have demonstrated that red blood cells (RBCs) carry large quantities of free cholesterol in their membranes and play an essential role in reverse cholesterol transport (Ohkawa et al., 2020). We postulate that the membranes of white blood cells are similarly enriched by free cholesterol.

Free cholesterol is crucial for cellular structure and function (making up 10–45 mol% of cell membrane lipids) and plasma‐circulating lipoproteins. The surfaces of HDL and LDL particles of normolipidemic individuals comprise about 25% and ~40% mol% free cholesterol, respectively (Gillard et al., 2023). Additionally, studies on rabbits show that free cholesterol comprises 15% of cholesterol remnants (Ross & Zilversmit, 1977). Free cholesterol trafficking is influenced by its low solubility in water compared to phospholipids, so free cholesterol has a tendency to exchange at relatively high rates among phospholipid surfaces. This spontaneous rapid transfer significantly affects free cholesterol distribution and cytotoxicity, especially under high plasma free cholesterol conditions. Its accumulation in macrophages is crucial for developing foam cells and their subsequent death. Free cholesterol also diffuses to almost all tissues, and RBCs in particular (Liu et al., 2021).

The effects of free cholesterol on human macrophages have yet to be precisely investigated. Since reductions in the membrane‐free cholesterol levels can impact numerous cellular processes, elevated levels may similarly affect macrophage behavior. Increased membrane‐free cholesterol has been linked to macrophage retention in atherosclerotic lesions, promoting plaque progression (Grosheva et al., 2009; Nagao et al., 2007). We assume that similar enrichment of macrophages in adipose tissue is responsible for their increased presence in the adipose tissue of non‐obese individuals with elevated cholesterol levels, contributing to tissue‐specific and whole‐body inflammatory statuses. A series of in vitro experiments was conducted to examine how free cholesterol enrichment of the cell membrane affects macrophage properties. To achieve our goal, cholesterol was delivered to macrophages using methyl‐beta‐cyclodextrin (MβCD), which allows studying cholesterol effects without the confounding influence of apolipoprotein binding to its receptors (Qin et al., 2006). Additionally, we describe the potential effect of cholesterol remnants and LDL‐c levels on the proportion of pro‐inflammatory and anti‐inflammatory visceral adipose tissue macrophages in vivo.

## 2 METHODS

### 2.1 Living kidney donors

Between February 2020 and September 2022, 56 individuals were enrolled in the study after receiving comprehensive information about the kidney donation and transplantation procedures, including adipose tissue sampling during organ cleansing before transplantation. All participants provided informed consent, documented through forms that detailed the adipose tissue sampling process, and participated in interviews covering their medical history, diet, and significant cardiovascular risk factors. The present study was approved by the joint Ethics Committee of the Institute for Clinical and Experimental Medicine and Thomayer University Hospital, under the code number G‐19‐29, in compliance with the 1975 Declaration of Helsinki, as revised in 2000, and was conducted following the approved protocol.

### 2.2 Lipoprotein analysis

Blood samples were collected in the morning on the day of transplantation following a 12‐h fasting period. Separated sera were immediately frozen and stored at −80°C, with analysis performed in two portions (after sample 35 and at the study end). Cholesterol (04718917190), LDL‐C (04714423190), and HDL‐C (04713214190) fractions were analyzed using Roche Diagnostic kits. Remnant particle cholesterol fractions were determined as the difference between total cholesterol and the sum of LDL‐C and HDL‐C using the following formula: *Remnant cholesterol* [*mmol/L*] *= Total cholesterol* [*mmol/L*] *– LDL‐C* [*mmol/L*] *– HDL‐C* [*mmol/L*].

### 2.3 Adipose tissue analysis

**FIGURE 1:**
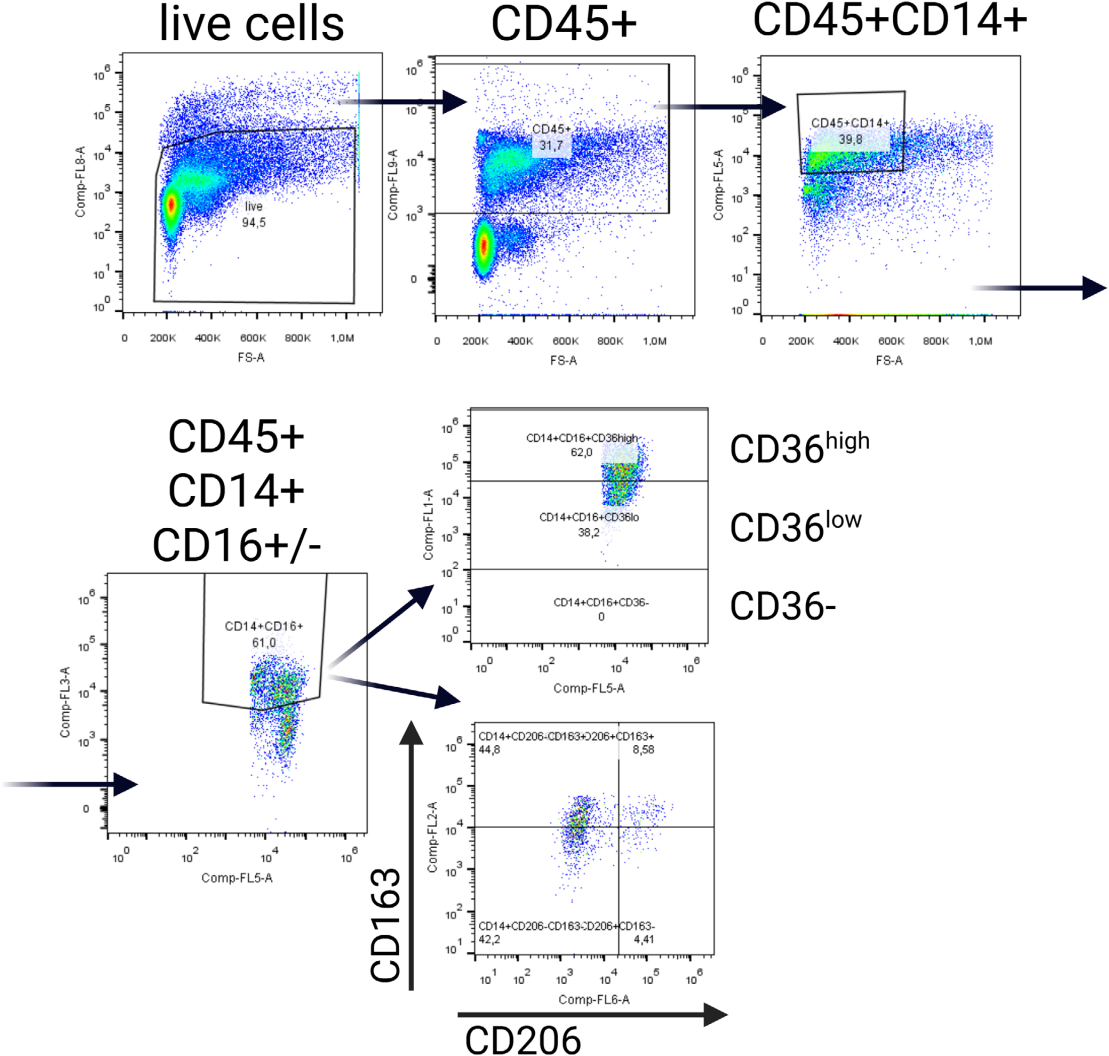
Example of SVF flow cytometric analysis. Live cells were first identified and gated using viability staining. Leukocytes were defined as CD45^+^ cells, and macrophages were further selected as CD45^+^CD14^+^ cells. These macrophages were then subdivided based on CD16 expression into CD16^−^ and CD16^+^ populations. Anti‐inflammatory macrophages were identified as CD45+CD14+CD16‐CD163+ cells, while pro‐inflammatory macrophages were defined as the CD45 + CD14 + CD16 + CD36^high^ subpopulation.

A visceral perirenal adipose tissue (VAT) sample was obtained intraoperatively, promptly cooled, and transferred to the laboratory. The adipose tissue processing method was extensively described previously (Kralova Lesna et al., 2015). The adipose tissue stromal vascular fraction (SVF) was isolated and analyzed after cleaning, dissection, and collagenase‐mediated disintegration (2 mg/mL) for 15 min at 37°C. The resulting homogenate was filtered through a 50 μm filter and subsequently centrifuged. The SVF underwent purification twice through resuspension. Final SVF samples were promptly analyzed using flow cytometry (Navios, Beckman Coulter, Brea, CA, USA). Only samples with viability exceeding 75%, as measured using Fixable Viability Dye eFluor™ 780 (Invitrogen, 65–0865‐18), were included for analysis. Analysis of cell surface antigens was performed as described. Briefly (Poledne et al., 2016), monoclonal antibodies and fluorochromes – CD14‐PC7 (Beckman Coulter, A22331), CD16‐ECD (Beckman Coulter, B49216), CD36‐FITC (Beckman Coulter, B49201), CD163‐PE (BioLegend, 326,506), CD206‐APC (BioLegend, 321,110), CD45‐Alexa Fluor 405 (R&D Systems, FAB1430V) – were utilized to identify various subsets of viable monocytes and adipose tissue macrophages. Each measurement was based on CD45^+^ cell counts ranging from 3175 to 449,000 per sample, with a median of 34,441 cells. The data were processed with FlowJo software (Becton, Dickinson & Company, Franklin Lakes, NJ, USA). The subsets were identified as previously described (Poledne et al., 2016): pro‐inflammatory metabolically activated (CD14 + CD16 + CD36^hi^) and anti‐inflammatory alternatively activated (CD14 + CD16‐CD163^+^) macrophages. A representative example of the gating strategy applied to visceral adipose tissue macrophages is presented in Figure 1.

### 2.4 Primary monocyte–macrophage cultures

Buffy coats from volunteer healthy donors, provided by the Blood Transfusion Service, Thomayer University Hospital, Prague, Czech Republic (approved by the Ethics Committee under permit No. 2591/23), were used. Peripheral blood mononuclear cells (PBMCs) were isolated using density gradient centrifugation with Ficoll‐Paque (Merck, GE17‐1440‐02) and centrifuged at 800 × rcf for 30 min. The PBMCs were collected, sedimented, and washed three times with PBS to remove contaminating platelets and then suspended in RPMI‐1640 Medium [RPMI supplemented with 10% heat‐inactivated fetal bovine serum, 1% Penicillin/Streptomycin and 1% GlutaMAX (Gibco, 35,050,061)]. The washed PBMCs were transferred onto 24‐well plates (Merck, CLS3526) (1.5 million cells/well) and allowed to adhere for 1 h before replacing the medium with one containing 50 ng/mL of recombinant human M‐CSF (PeproTech, 300–25). Cells were cultured for 6 days in a serum‐free medium replaced every 2 days with an M‐CSF‐containing medium to maintain constant M‐CSF levels. In total, macrophages derived from 30 human donors were investigated.

### 2.5 Preparation of cholesterol complexes

According to a previously published work by Christian et al. (Christian et al., 1997), we prepared complexes of cholesterol‐chelated (Merck, C3045) methyl‐β‐cyclodextrin (Merck, C4555) (as “water‐soluble cholesterol” with a molar ratio of 1:8 cholesterol/MβCD). To evaluate cholesterol incorporation, radiolabeled cholesterol [^14^C] (ARC, ARC‐0857, specific activity 0.1 μCi/mL) was incorporated at a final 0.024 μCi/mol specific activity. Unless otherwise specified, a cholesterol concentration of 242 μg/mL was utilized to analyze cell surface receptors and in gene expression analysis.

### 2.6 Cholesterol influx assay

To assess the uptake of free cholesterol by macrophages and macrophage enrichment with free cholesterol, we conducted experiments using M‐CSF‐polarized macrophage cultures. These cultures were incubated under defined conditions on the day of the experiment, focusing on evaluating the impact of time, concentration, and temperature. Prior to incubation, the macrophage cultures were washed with PBS and, subsequently, cholesterol complexes containing radiolabeled cholesterol at the desired concentration in the RPMI medium were added to the macrophages to initiate incubation.

Following the incubation period, the supernatants were removed, and the cell monolayers were washed twice with ice‐cold PBS. The cells were then frozen for 1 h, defrosted, and lysed in isopropanol‐hexane (2:3) for another hour. Aliquots of the lysates were centrifuged at 400 rcf and then evaporated in scintillation vials. Next, the scintillation liquid (Rotiszint eco plus, Roth, 1P18.2) was added to the aliquots for liquid scintillation counting.

The extent of macrophage enrichment with free cholesterol was determined based on the radioactivity of the lysed cells. All samples were analyzed in six replicates using a liquid scintillation analyzer (Tri‐Carb 2900TR; PerkinElmer, Waltham, MA, USA) and the presented data are the means of the six replicates. The free cholesterol enrichment in macrophages was measured in samples taken at intervals ranging from 5 to 90 min post incubation with radiolabeled cholesterol complexes.

To assess how macrophage enrichment with free cholesterol affects the expression of surface markers, human M‐CSF‐polarized macrophages were exposed to different treatments on the day of the experiment; they were either incubated with cholesterol complexes (242 ug/mL), LPS (Merck, L2654) (100 ng/mL) or with no supplement. The latter treatment (with no media supplement) served as a negative control, while incubation with LPS acted as a positive control based on its strong pro‐inflammatory effect. These controls helped to evaluate the impact of free cholesterol enrichment. After a 90‐min incubation, the supernatants were removed, and the cell monolayers were washed twice with ice‐cold PBS. The macrophages were detached from the Nunc™ UpCell™ (Thermo Scientific, 174,899) dishes at 4°C without using enzymes. Following the washes, the cells were stained for flow cytometry analysis.

The cells used for the gene expression evaluation were grown on adherent dishes under the same conditions as those used for surface marker analysis. On the day of the experiment, after a 90‐min incubation, the supernatants were discarded and the cell monolayers were washed twice with ice‐cold PBS. Total cellular RNA was then extracted using TRIzol Reagent extraction (Molecular Research Center, TR118), quantified using a NanoDrop ND‐1000 spectrophotometer (NanoDrop Technologies, Wilmington, DE, USA) and frozen for further analysis.

### 2.7 Flow cytometry and antibodies for macrophage analyses

Cell surface antigens for M‐CSF‐polarized macrophages, which were incubated on the day of the experiment, were analyzed in the same manner as described in the above paragraph. Each measurement was based on cell counts ranging from 1002 to 10,182 per sample, with a median of 3032 cells. The data were processed using FlowJo software.

### 2.8 Gene expression analysis

Following the previously described quantitative real‐time PCR procedures (Čejková et al., 2017), the mRNA expression levels of genes encoding proteins associated with macrophage polarization and cholesterol balance were quantified after 90 min of incubation of M‐CSF‐polarized macrophages with free cholesterol or LPS or in media without supplementation. The RNA from each sample was converted to cDNA; 100 ng of total RNA was used for reverse transcription. After treatment with DNase I (Merck, AMPD1‐1KT) to eliminate DNA contamination, cDNA synthesis was performed according to the High Capacity RNA‐to‐cDNA Master Mix kit (Applied Biosystems, 4,388,950) manufacturer's instructions. Quantitative RT‐PCR analyses were conducted in triplicate. Gene expression levels were measured using the Corbett Life Science Rotor‐Gene 3000 cycler (Qiagen, Venlo, The Netherlands) with HOT FIREPol® EvaGreen® qPCR Mix Plus (ROX) (Solis Bio Dyne, 08–24‐00001). The nucleotide sequences of the primer pairs used in the study were as shown below. The following primer pairs for TNFα (tumor necrosis factor α, NM_000594.4; forward: CCTCTCTCTAATCAGCCCTCTG; reverse: GAGGACCTGGGAGTAGATGAG), MCP‐1a (CCL3, C‐C motif chemokine ligand 3, NM_002983.3; forward: AGTTCTCTGCATCACTTGCTG; reverse: CGGCTTCGCTTGGTTAGGAA), CD36 (CD 36 molecule, NM_000072.3; forward: GGCTGTGACCGGAACTGTG; reverse: AGGTCTCCAACTGGCATTAGAA), and ACAT1 (acetyl‐CoA acetyltransferase 1, NM_000019.4; forward: AAGGCAGGCAGTATTGGGTG; reverse: ACATCAGTTAGCCCGTCTTTTAC) were used. Beta‐2‐microglobulin (B2M, NM_004048.2; forward: TCTCTCTTTCTGGCCTGGAG; reverse: AATGTCGGATGGATGAAACC) served as the endogenous control for normalization. All primers were synthesized in GENERI BIOTECH (Hradec Kralove, Czech Republic). Relative gene expression was determined by the delta–delta Ct method.

### 2.9 Statistical analysis

Descriptive statistics for the living kidney donor group analysis and the cholesterol influx assay data are presented as medians with their minima and maxima. For some variables (as shown together with their respective results), the original values were log‐transformed before analyses to achieve approximate normality of the distributions. The plots (with the exception of the correlation scatter plots) present the point estimates together with two‐sided 95% confidence intervals constructed using the normal approximation. To assess the associations between the pro‐inflammatory and anti‐inflammatory visceral adipose tissue macrophage subpopulations and various types of serum cholesterol lipoproteins, we used the Pearson correlation coefficient and the coefficient of determination. Between‐group and between‐time point differences for all variables (cholesterol influx assay) were tested using t‐tests. Multivariable linear models were employed to investigate the effects of temperature and dose on free cholesterol loading and free cholesterol enrichment of macrophage cultures. For all the tests, the threshold of *p* < 0.05 for statistical significance was used. The p‐values are presented without multiple testing adjustment with the exception of the gene expression tests, which were adjusted by the Holm method as a single family of tests. Correlation analysis was performed using biostatistical GraphPad Prism software, version 6 (GraphPad Software, San Diego, CA, USA). The remaining analyses were performed using R software version 4.3.2 (R Foundation for Statistical Computing, Vienna, Austria).

## 3 RESULTS

### 3.1 The effect of serum cholesterol lipoproteins on adipose tissue macrophage surface markers

The characteristics of the study group consisting of 56 living kidney donors are summarized in Table 1. Twenty‐two individuals had hypercholesterolemia (>5 mM), nine were treated with statins, and seventeen received antihypertensive drugs. Additionally, the BMI was over 30 in four cases.

**TABLE 1:** Characteristics of the living kidney donors (male, *n* = 21; female, *n* = 35).

Characteristics	*N*	Median	Min	Max
Age (years)	56	52	35	75
BMI (Kgm^−2^)	56	21.1	20.2	31.2
Total cholesterol (mmol/L)	56	4.75	2.22	7.70
HDL‐c (mmol/L)	56	1.19	0.59	1.80
Non‐HDL‐c (mmol/L)	56	3.35	1.33	6.52
Triacyglycerides (mmol/L)	56	1.57	0.57	3.78

**FIGURE 2:**
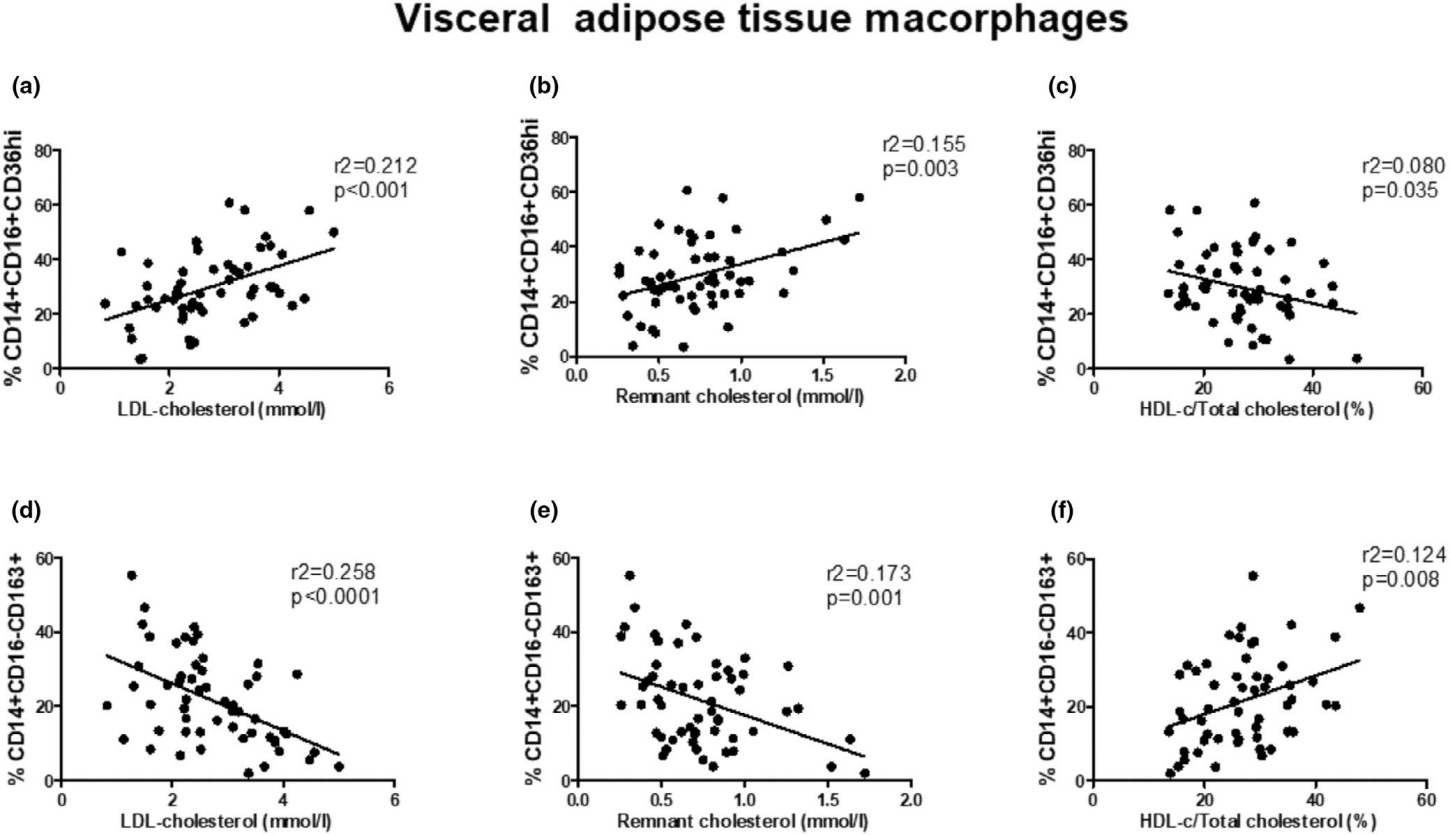
The relationship between visceral adipose tissue macrophages and plasma lipoprotein levels in 56 living kidney donors. Correlation between pro‐inflammatory CD14 + CD16 + CD36^high^ macrophages and (a) LDL‐C (mmol/L), (b): Remnant cholesterol (mmol/L), (c) HDL‐cholesterol/total cholesterol %). Correlation between anti‐inflammatory CD14 + CD16‐CD163+ macrophages and (d) LDL‐C (mmol/L), (e) remnant cholesterol (mmol/L), (f) HDL‐cholesterol/total cholesterol (%). The correlation plots include the coefficients of determination (*r* ^2^) and the *p*‐values.

The relationship between serum lipoproteins and the percentage of specific macrophage subsets in VAT is depicted as a series of scatter plots (Figure 2). A positive correlation was found between LDL‐C levels and the percentage of pro‐inflammatory CD14 + CD16 + CD36^high^ macrophages, with a coefficient of determination r^2^ of 0.212 and a statistically highly significant value (*p* < 0.001). Individuals with LDL‐C levels below 2 mmol/L exhibited a nearly threefold lower proportion of pro‐inflammatory macrophages than those with concentrations around 3 and 4 mmol/L. We calculated the cholesterol concentration in remnant particles to gain deeper insights into the role of serum cholesterol in macrophage polarization. There was also a correlation between remnant cholesterol levels and the percentage of CD14 + CD16 + CD36^high^ macrophages, with an r^2^ of 0.155 and *p* = 0.003. A negative correlation was revealed between the HDL/total cholesterol ratio and the percentage of CD14 + CD16 + CD36^high^ macrophages, with an r^2^ of 0.080 and *p* = 0.035.

Further, an analysis of the correlation between the anti‐inflammatory alternatively activated macrophages (CD14 + CD16‐CD163+) and VAT was performed. The strongest negative correlation was observed between LDL‐C levels and the percentage of CD14 + CD16‐CD163+ macrophages, with an r^2^ of 0.258 and *p* < 0.0001. The relationship between remnant cholesterol levels and the percentage of anti‐inflammatory macrophages correlated negatively, with an r^2^ of 0.173 and *p* = 0.001. A positive correlation was observed between the HDL/total cholesterol ratio and the rate of the subpopulation of CD14 + CD16‐CD163+ macrophages, with an r^2^ of 0.124 and *p* = 0.008.

### 3.2 Free cholesterol transport to human macrophages

We investigated the efficiency of free cholesterol loading into human macrophages through the cell membrane using MβCD‐cholesterol complexes under different conditions.

**FIGURE 3:**
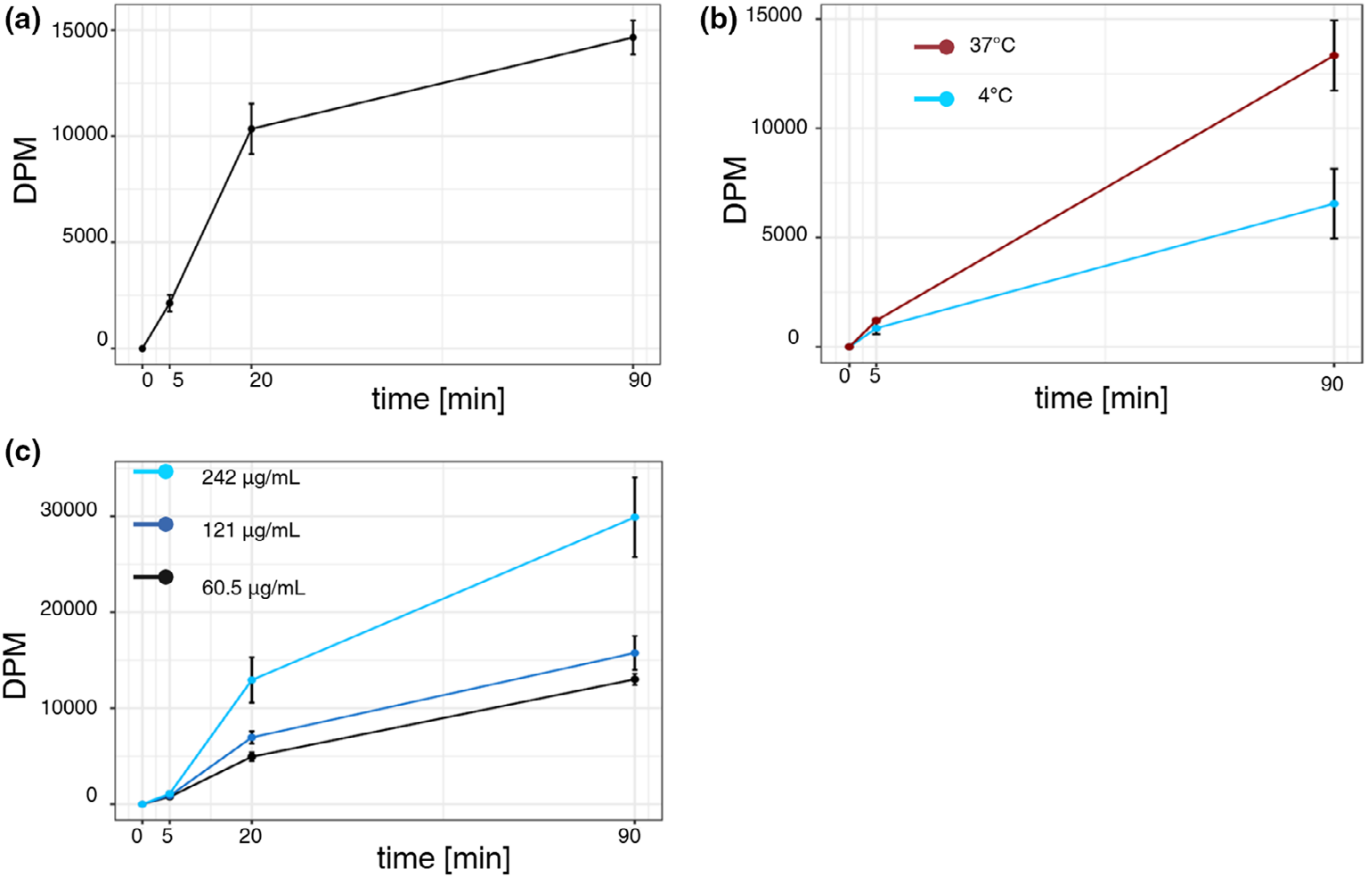
Transport of free cholesterol from MβCD‐cholesterol complexes to human macrophage cultures under various conditions. DPM represents transferred free cholesterol from complexes into macrophage cultures, as determined from cellular extracts. A: DPM after incubation for up to 90 min. Each data point represents an average of twelve measurements (six per subject). B: DPM after incubation for up to 90 min at reduced (4°C) and normal (37°C) temperatures. Each data point represents an average of four measurements. C: DPM after incubation for up to 90 min at different initial free cholesterol concentrations in the incubation medium. Each data point represents an average of six measurements. In all experiments, macrophages were incubated with radiolabeled cholesterol [14C] at a final specific activity of 0.024 μCi/mol. The incubation medium contained 242 μg/mL of total cholesterol. Each data point is shown with a two‐sided 95% confidence interval.

The amount of cholesterol successfully incorporated into the macrophages was found to increase with exposure to MβCD‐cholesterol complexes for up to 90 min at 37°C. Figure 3a shows the relationship between time (in minutes) and DPM (disintegrations per minute), which represents the incorporation of free cholesterol into macrophage cultures. Macrophage enrichment with free cholesterol was determined from cellular extracts at 5, 20, and 90 min, based on the detection of specific activity incorporated [^14^C]. Each data point represents an average from two subjects (6 measurements per subject).

The amount of free cholesterol incorporated into macrophage cell cultures changes depending on the temperature during incubation. The cells were exposed to free cholesterol complexes for 5 to 90 min at either 37°C or 4°C to see the variability in free cholesterol loading. Figure 3b illustrates the relationship between time and DPM under the above temperature conditions. At both 37°C and 4°C, there was an upward trend over time indicating that macrophage enrichment with free cholesterol increases at both temperatures. In a multi‐linear model (total of 4 data points used), both the overall increase (*p* = 0.001) and the difference in increase based on temperature (interaction term for time and temperature, *p* = 0.01) were statistically significant.

Increasing the concentration of free cholesterol in the incubation medium significantly increased the amount of free cholesterol in macrophage cultures. Figure 3c shows the transfer of free cholesterol from complexes to macrophage cultures over time (measured as DPM). In a multi‐linear model (total 9 data points used), the difference in the increase in transport based on the initial concentration (interaction term for time and concentration) was statistically significant (*p* = 0.002). This indicates that higher concentrations in the incubation medium result in greater amounts of free cholesterol transferred to macrophage cultures, leading to higher levels of free cholesterol in the cells over time. Based on the DPM observed and free cholesterol concentrations in the incubation medium, we calculated that the amounts of free cholesterol transported to a single well (containing 450,000 macrophages) were 13.5 μg with the highest initial concentration, 7.1 μg with intermediate concentration, and 5.9 μg with the lowest one.

### 3.3 Free cholesterol loading converges gene expression in M‐CSF‐polarized macrophages to LPS‐associated responses

**FIGURE 4:**
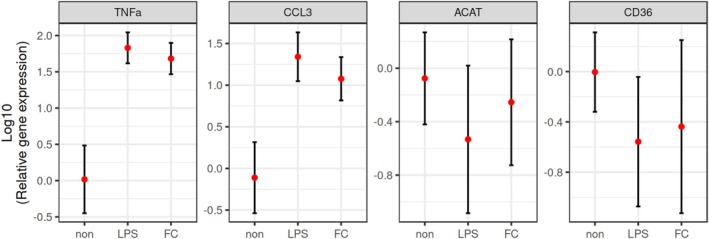
The relative gene expression of human M‐CSF‐polarized macrophage cultures after 90 min of incubation in media containing free cholesterol (FC). The genes analyzed included those of tumor necrosis factor‐alpha (TNFa), chemokine (C‐C motif) ligand 3 (CCL3), acetyl‐CoA acetyltransferase (ACAT), and cluster of differentiation 36 (CD36). The original values were log‐transformed before analyses. “Non” indicates 90 min of incubation in media without supplementation, while “LPS” indicates 90 min of incubation in media with LPS (100 ng/mL). The red points represent the means of 9 samples (each sample per subject), and each point is estimated along with a two‐sided 95% confidence interval.

We conducted a gene expression analysis to identify changes in the M‐CSF‐polarized macrophage cultures in the setting of free cholesterol overaccumulation. Free cholesterol exposure significantly upregulated gene expression of the pro‐inflammatory markers *TNFα* (58‐fold increase, *p* < 0.001) and *MCP‐1a* (CCL3) (15.7‐fold increase, p < 0.001) compared to the negative control. This response was comparable to the effect of pro‐inflammatory stimulation by LPS controls for both genes (both p < 0.001). However, the mRNA expression levels of genes associated with lipid uptake (*CD36*) and intracellular cholesterol trafficking (*ACAT1*) did not show any significant differences across all three incubation media conditions. The expression patterns of all genes *(TNFα, MCP‐1a (CCL3), ACAT1, and CD36)*, analyzed as the means of folds after log‐transformation of nine samples (one sample per subject), along with two‐sided 95% confidence intervals, are shown in Figure 4.

### 3.4 Cholesterol influx assay and macrophage surface marker changes

**FIGURE 5:**
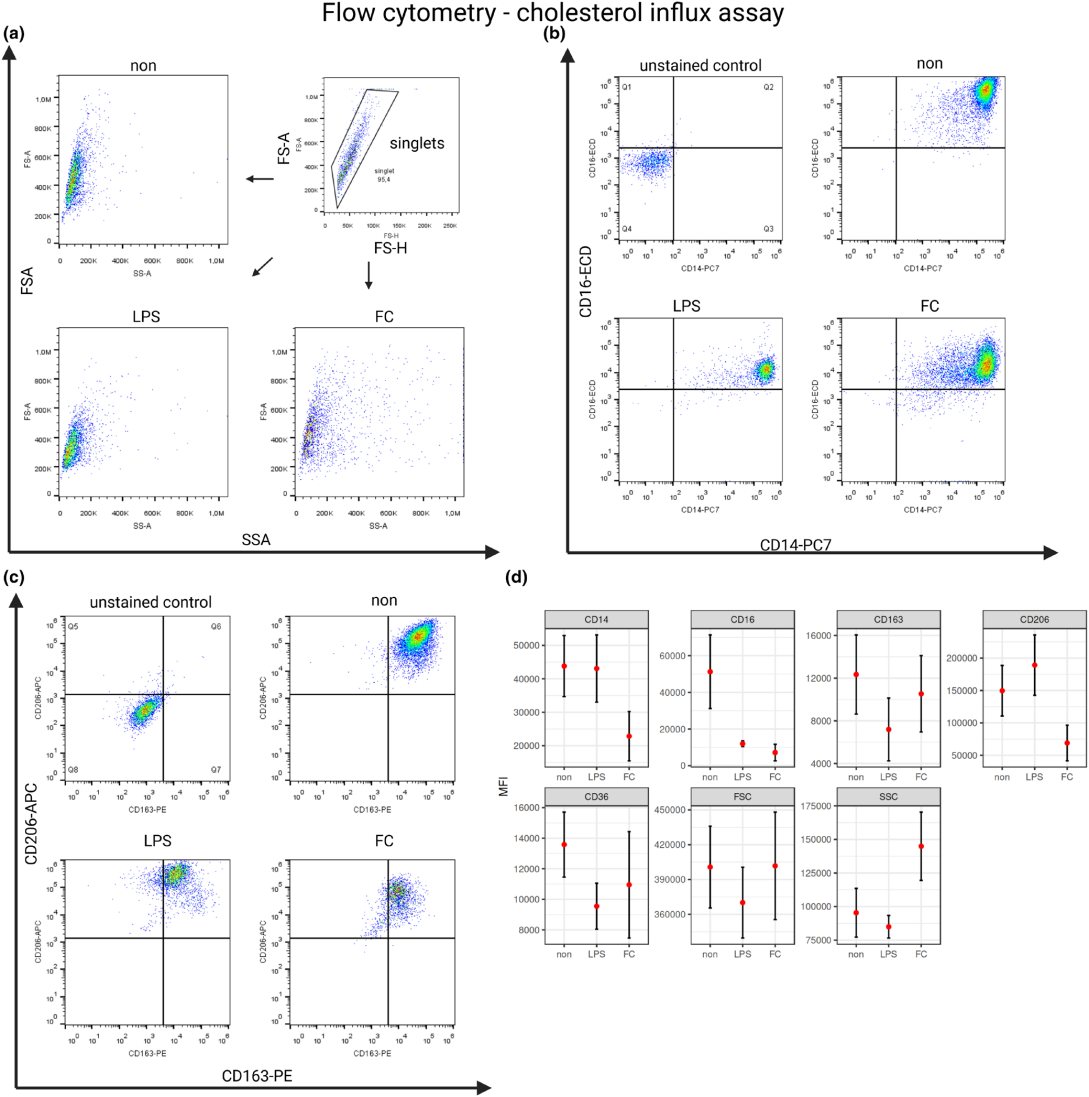
The surface marker expression in human M‐CSF–polarized macrophage cultures after 90 min of incubation with free cholesterol (FC). (a) The gating strategy identifies single cells, followed by size (FSC‐A) vs. granularity (SSC‐A) plots for each condition (non, LPS, FC). (b–c) Flow cytometry dot plots showing surface marker expression (CD14, CD16, CD163, CD206) in macrophage cultures incubated for 90 min under three conditions: No supplement (non), LPS (100 ng/mL, positive control), and free cholesterol (FC). Unstained controls are included for reference. (d) Median Fluorescence Intensity (MFI) values for CD14, CD16, CD163, CD206, CD36, and scatter parameters (FSC, SSC). Red points indicate the mean MFI of 7 biological replicates (one per donor), with two‐sided 95% confidence intervals.

Human M‐CSF‐polarized macrophage cultures were incubated for 90 min in free cholesterol‐containing media. Next, they were analyzed by flow cytometry to possibly identify any changes in their surface markers due to free cholesterol overaccumulation in the macrophage cell membrane. Compared to the negative control, the macrophage cultures shifted towards a pro‐inflammatory response. The expression of CD206 (*p* = 0.02), a marker typically associated with anti‐inflammatory polarization, was significantly decreased. The CD16 receptor expression also significantly decreased (p = 0.02) compared to the negative control, similar to the response observed with the positive LPS control compared to the negative control (*p* = 0.04). Additionally, there was a dramatic increase in granularity (side scatter) compared to both controls (*p* < 0.001 for both). The medians of fluorescence intensity for CD14, CD16, CD36, CD163, CD206, side and forward scatter counts, as the means of 7 samples (each sample per subject), along with two‐sided 95% confidence intervals, are shown in Figure 5.

## 4 DISCUSSION

Our research highlights the importance of cholesterol‐induced activation of pro‐inflammatory macrophages. We observed a connection between the polarization status of macrophages in adipose tissue and the plasma lipoprotein levels in both healthy men and women. Specifically, higher plasma cholesterol levels were associated with an increased proportion of pro‐inflammatory macrophages in visceral adipose tissue. Additionally, our experiments with in vitro M‐CSF‐polarized macrophages demonstrated pro‐inflammatory changes associated with the incorporation of free cholesterol. The pro‐inflammatory changes detected in macrophages both in vivo and in vitro are thought to be driven by cholesterol overaccumulation at the molecular level, as recently reported. Cholesterol overaccumulation in the cell membrane activates cholesterol trafficking from the cell membrane to the endoplasmic reticulum via the protein Aster‐B (Ferrari et al., 2020), which further promotes NLRP3 inflammasome activation (Yalcinkaya et al., 2024). This response was general to increasing cholesterol content in macrophage cell membranes, as demonstrated through various methods of raising cellular cholesterol (Yalcinkaya et al., 2024). These findings suggest a new mechanism to explain how dyslipidemia involving increased levels of cholesterol‐rich lipoproteins and decreased levels of cholesterol efflux promoting HDL can lead to pro‐inflammatory activation of macrophages and eventually to the development of an inflammatory disorder, atherosclerosis.

Our previous study identified specific markers for two types of macrophages: pro‐inflammatory metabolically activated macrophages (CD14 + CD16 + CD36^high^) and anti‐inflammatory alternatively activated macrophages (CD14 + CD16‐CD163+). We found that pro‐inflammatory macrophages had a strong positive correlation with non‐HDL cholesterol levels, while anti‐inflammatory macrophages showed a negative correlation with these levels (Poledne et al., 2016).

In our most recent research, we further analyzed these relationships using the same pro‐inflammatory and anti‐inflammatory markers, given the absence of more suitable marker combinations that would better reflect the inflammatory status and its association with circulating lipoproteins. The results revealed a positive correlation between plasma remnant cholesterol levels and the presence of pro‐inflammatory macrophages. In contrast, a negative association was observed between remnant cholesterol levels and the proportion of anti‐inflammatory macrophages. A similar association was found for LDL cholesterol levels, with even stronger correlations observed. For individuals with LDL‐C concentrations around 2 mmol/L, the ratio of pro‐inflammatory to anti‐inflammatory macrophages is approximately 2:3; however, at concentrations of 4 mmol/L, this ratio shifts markedly to 3:1, indicating a substantial alteration in the pro‐inflammatory status of adipose tissue. These findings suggest that individuals with lower intravascular LDL‐C concentrations may experience accelerated polarization towards an anti‐inflammatory phenotype within adipose tissue, thus emphasizing the critical role of adipose tissue macrophages in modulating systemic inflammatory status. These findings support the long‐held understanding that high plasma levels of LDL particles are a significant risk factor for atherosclerosis (Goldstein & Brown, 1973).

Given the link between lipoproteins and circulating monocytes, monocyte‐derived macrophages in visceral and perivascular adipose tissue (Coen et al., 2010; Hjuler Nielsen et al., 2015), and systemic inflammation (Poledne et al., 2022)—together with evidence of a higher incidence of cardiovascular disease (CVD) in individuals with an increased ratio of free‐to‐esterified cholesterol in circulating lipoproteins (Bagheri et al., 2018; Feng et al., 2020), and the previously documented induction of pro‐inflammatory genes (O'Rourke et al., 2022) and inflammasome activation (Yalcinkaya et al., 2024) in macrophages due to the accumulation of cholesterol crystals (of which free cholesterol is the principal structural component) in the subendothelial space—we next developed an in vitro assay to investigate how free cholesterol directly influences macrophage activation.

Animal cells tightly control cholesterol levels in their cell membranes, where most of the cell's total cholesterol is located (Goldstein & Brown, 2001). Various experiments have demonstrated that the dynamic cholesterol pool in the cell membrane is accessible for both cholesterol integration into cells (via vesicular and non‐vesicular mechanisms) and its export from cells. An accessible free cholesterol pool is likely located in the outer membrane leaflet (Das et al., 2014). MβCD cholesterol complexes in our assay have been shown to effectively and rapidly enrich human macrophages with cholesterol. The loading process is exceptionally rapid, occurring within minutes to hours. Through a series of experiments, we demonstrated that the uptake of free cholesterol by human PBMCs from these complexes depends on the temperature, time, and concentration. Regarding the observed temperature dependency, we speculate that low temperatures increase membrane rigidity (Los & Murata, 2004) and alter the properties of the cell membrane (Goldenthal et al., 1984; Smith et al., 2012), thereby reducing the efficiency of simple diffusion of free cholesterol. Based on evidence from fluorescence staining (Buwaneka et al., 2021; Qin et al., 2006), which confirms cell membrane enrichment and the ratio of free‐to‐esterified cholesterol within the first two hours of incorporation (Christian et al., 1997; Kruth et al., 1995), and based on data obtained in our assay, we assume that cholesterol enrichment predominantly occurs in the cell membrane and its surface‐connected intracellular compartments.

The present study focuses on inflammatory activation responses of cholesterol‐loaded human monocyte‐derived macrophages resulting from their exposure to LPS or free cholesterol after differentiation in M‐CSF. To avoid the generation of serum heterogeneous populations, the monocytes were matured in serum‐free media at a given concentration of M‐CSF (Lappalainen et al., 2021). To investigate the effects of free cholesterol loading on the expression of pro‐ and anti‐inflammatory genes in cultured human M‐CSF‐polarized macrophages, we used our cholesterol assay and compared the levels of gene expression in cholesterol‐loaded macrophages, LPS‐activated macrophages, and non‐stimulated macrophages. We found that the generated macrophages were polarized into the pro‐inflammatory response by activation with LPS and responded with typical upregulation of pro‐inflammatory genes *(TNFA, CCL3)* (Lawlor et al., 2021). While macrophages after free cholesterol loading similarly upregulated both genes, the *CD36* gene, which controls lipid uptake, and the cholesterol esterification enzyme *ACAT* displayed variable responses. Thus, the levels of both selected pro‐inflammatory genes were increased by the LPS and free‐cholesterol activation, thus rendering the free cholesterol‐loading macrophages more pro‐inflammatory than non‐activated macrophages.

To confirm the effect of free cholesterol loading on macrophages response, we used flow cytometry. Accumulation of cholesterol in macrophages leads to the formation of an increasingly compartmentation‐like structure. Upshifted Signals detected from side scatter channels of cholesterol‐loaded macrophages confirmed that the incubation with cholesterol had led to structural changes in macrophages. Similar findings were previously described after 2 h of incubation of macrophages with microcrystalline cholesterol (Kruth et al., 1995).

Additionally, the expression of surface markers on M‐CSF‐polarized macrophages after free cholesterol loading revealed the complexity of macrophage polarization extending beyond the simple M1/M2 paradigm. The complexity was highlighted by the different responses between LPS activation and cholesterol loading in the expression of the analyzed surface markers such as CD206 and CD163. The CD206 and CD163 surface receptors are markers of the anti‐inflammatory macrophage status; thus, their decreased expression is associated with pro‐inflammatory changes. In our results, cholesterol loading downregulated CD206 without causing significant changes in CD163 compared to non‐stimulated cells. Further complexity in macrophage responses was observed when comparing LPS activation. LPS activation significantly downregulated CD163 expression, while the pro‐inflammatory marker CD206 was upregulated following LPS activation. Nielsen et al. observed similar patterns of CD163 and CD206 regulation within the first hours of incubation with LPS (Nielsen et al., 2019). A possible explanation could relate to the different properties and roles of these receptors. CD206 is involved in macrophage clearance of endogenous molecules, antigen presentation, and the modulation of cellular activity and trafficking (Nielsen et al., 2019). Macrophages expressing CD206 exhibit high phagocytic activity (Chinetti‐Gbaguidi et al., 2011). CD163, on the other hand, is a scavenger receptor primarily responsible for the removal of toxic hemoglobin from circulation and (Nielsen et al., 2019), compared to CD206‐expressing macrophages, may be more involved in lipid and cholesterol uptake (Chinetti‐Gbaguidi et al., 2011). Moreover, the two receptors differ in the time required for their release from the macrophage membrane in soluble form (Nielsen et al., 2019). In our in vitro assay, we further observed CD16 downregulation after 90 min of LPS activation and cholesterol loading compared to unstimulated macrophages. This contrasts with previous observations, where the proportion of CD16^+^ macrophages increases with rising cholesterol levels. These unexpected results are likely due to the addition of M‐CSF, which markedly increases basal CD16 expression (Ambarus et al., 2012), as represented in our assay by unstimulated macrophages.

## 5 CONCLUSION

This study demonstrates that both LDL‐C and remnant cholesterol are positively associated with the proportion of pro‐inflammatory macrophage subpopulations in visceral adipose tissue, even in clinically healthy individuals with normal plasma lipoprotein levels. This relationship may contribute to whole‐body low‐grade inflammation, a key process in the pathogenesis of atherosclerosis. Furthermore, our in vitro findings show that free cholesterol, upon short‐term incorporation into macrophage membranes, induces the expression of pro‐inflammatory genes and alters the pattern of surface receptors in a pro‐inflammatory direction. These results suggest free cholesterol as a biologically active lipid component that may be responsible for the pro‐inflammatory potential of both LDL‐C and remnant particles.

## FUNDING INFORMATION

Supported by (1) the project National Institute for Research of Metabolic and Cardiovascular Diseases (Programme EXCELES, ID Project No. LX22NPO5104)–Funded by the European Union–Next Generation EU, and (2) the Ministry of Health of the Czech Republic–RVO (“Institut klinické a experimentální medicíny–IKEM, IČ 00023001”).

## CONFLICT OF INTEREST STATEMENT

The authors have nothing to disclose.

## ETHICS STATEMENT

The study was approved by the Joint Ethics Committee of the Institute for Clinical and Experimental Medicine and Thomayer University Hospital. Samples from 56 living kidney donors were collected under approval code G‐19‐29, and buffy coats were obtained under permit No. 2591/23. All experimental procedures were conducted in accordance with the approved ethical protocols. All participants received written information about the purpose and procedures of the study and provided written informed consent prior to data collection. Written informed consent was also obtained for the publication of anonymized patient information included in this article.

## Data Availability

The data that support the findings of this study are available on request from the corresponding author.
