# Correction to “Human macrophage pro‐inflammatory polarization in response to free cholesterol and cholesterol remnants”

**DOI:** 10.14814/phy2.70570

**Published:** 2025-09-23

Karel, P., Barbora, M., Hana, B., Jan, M., Libor, J., Jiri, F., Sona, K., Ivana, K. L., & Rudolf, P. (2025). Human macrophage pro‐inflammatory polarization in response to free cholesterol and cholesterol remnants. *Physiological Reports*, 13, e70367. https://doi.org/10.14814/phy2.70367.

In the publication, the given names and surnames of all authors were mistakenly reversed.

The list below gives the authors' names in their correct forms, with first names and surnames clearly indicated:

**Table:** 

First name	Surname
Karel	Paukner
Barbora	Muffova
Hana	Bartuskova
Jan	Mareš
Libor	Janousek
Jiri	Fronek
Sona	Kauerova
Ivana	Kralova Lesna
Rudolf	Poledne

We apologize for this error.

